# Structural characterization of the carbohydrate-binding module of NanA sialidase, a pneumococcal virulence factor

**DOI:** 10.1186/s12900-015-0042-4

**Published:** 2015-08-20

**Authors:** Lei Yang, Helen Connaris, Jane A. Potter, Garry L. Taylor

**Affiliations:** Biomedical Sciences Research Complex, University of St Andrews, St Andrews, Fife KY16 9ST UK

## Abstract

**Background:**

*Streptococcus pneumoniae* Neuraminidase A (NanA) is a multi-domain protein anchored to the bacterial surface. Upstream of the catalytic domain of NanA is a domain that conforms to the sialic acid-recognising CBM40 family of the CAZY (carbohydrate-active enzymes) database. This domain has been identified to play a critical role in allowing the bacterium to promote adhesion and invasion of human brain microvascular endothelial cells, and hence may play a key role in promoting bacterial meningitis. In addition, the CBM40 domain has also been reported to activate host chemokines and neutrophil recruitment during infection.

**Results:**

Crystal structures of both apo- and holo- forms of the NanA CBM40 domain (residues 121 to 305), have been determined to 1.8 Å resolution. The domain shares the fold of other CBM40 domains that are associated with sialidases. When in complex with α2,3- or α2,6-sialyllactose, the domain is shown to interact only with the terminal sialic acid. Significantly, a deep acidic pocket adjacent to the sialic acid-binding site is identified, which is occupied by a lysine from a symmetry-related molecule in the crystal. This pocket is adjacent to a region that is predicted to be involved in protein-protein interactions.

**Conclusions:**

The structural data provide the details of linkage-independent sialyllactose binding by NanA CBM40 and reveal striking surface features that may hold the key to recognition of binding partners on the host cell surface. The structure also suggests that small molecules or sialic acid analogues could be developed to fill the acidic pocket and hence provide a new therapeutic avenue against meningitis caused by *S. pneumoniae*.

## Background

*Streptococcus pneumoniae* is a human pathogen responsible for respiratory tract infections, septicaemia and meningitis. Several virulence factors contribute to colonization and early infection processes [1]. Sialidases from pathogenic bacteria are considered as key virulence factors, as they remove sialic acid from host cell surface glycans, unmasking certain receptors to facilitate bacterial adherence and colonization. All *S. pneumoniae* clinical isolates investigated to date possess prominent sialidase activities. Three sialidases, NanA, NanB and NanC, are encoded by *S. pneumoniae* genomes. A study of sialidase genes in clinical pneumococcal isolates identified *nanA, nanB* and *nanC* to be present in 100 %, 96 % and 51 % of these strains, respectively [2]. Pneumococcal strains with knockouts of *nanA* or *nanB,* and studied in mouse models, show that both proteins are essential to *S. pneumoniae* infection of the respiratory tract and sepsis [3]. NanA, specifically, has been shown to play an important role in host-pneumococcal interactions in the upper respiratory tract [4, 5], and is involved in biofilm formation [6]. It has also been shown to desialylate competing bacteria such as *Neisseria meningitidis* and *Haemophilus influenzae,* potentially giving *S. pneumoniae* an advantage in shared bacterial niches [7]. Furthermore, the NanA from *S. pneumoniae* has also been shown to promote inflammation by disrupting sialic acid based recognition of CD24 by SiglecG in mice (the equivalent of SIGLEC10 in humans) [8].

**Fig. 1:**
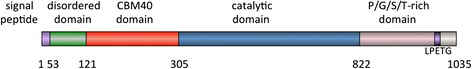
Schematic of the NanA domains

From amino acid sequence comparison of bacterial sialidases, NanA is modular by nature and its domain organisation is similar to other known bacterial sialidases (Fig. 1). The enzyme contains a catalytic domain flanked by an N-terminal carbohydrate-binding domain (CBM) that is downstream of a signal sequence, followed by a region of predicted disorder. At the C-terminus, there is a region rich in proline, glycine, threonine and serine containing a sequence of 20 amino acids repeated three times contiguously followed by an LPXTG anchor sequence. Subsequent analysis of multiple pneumococcal strains showed that the *nanA* gene is highly diverse, mainly in truncations in the C-terminal region, but that the N-terminal CBM and catalytic domain are conserved [9]. Two studies have reported the importance of NanA in allowing *S. pneumoniae* to adhere to and invade the blood–brain-barrier (BBB), through the use of human brain microvascular endothelial cells (hBMECs). In particular the N-terminal CBM domain (described in the study as a laminin G-like domain) was found to be the critical determinant of this event [10, 11]. Both of these studies showed that the catalytic activity of NanA only played a minor role in the adhesion/invasion event, with one study showing that the N-terminal CBM was also involved in the induction of neutrophil chemo-attractants IL-8, CXCL-1 and CXCL-2 [11].

We recently cloned the N-terminal CBM domain of NanA (residues 121–305) and carried out a glycan array screen that showed the domain binds to sialic acid [12], and as such is a Family 40 CBM as defined in the CAZY database [13]. We have also engineered multivalent forms of this domain, designed to adhere with high affinity to sialic acid receptors in the respiratory tract, and have shown that they prevent infection from influenza viruses in a mouse model. Preliminary analysis of immunomodulators during this influenza study supports the ability of this domain to stimulate the immune system in mice, specifically IL-1β, MIP-2 (the mouse homolog of IL-8), IFN-γ and TNF-α [12].

To date, only the catalytic domain of NanA has been studied structurally, and is the subject of small molecule inhibitor studies [14–16]. Here we describe the crystal structure of the *S. pneumoniae* NanA CBM40 domain, hereafter named *Sp*CBM. Crystal structures of *Sp*CBM, complexed with α2,3-sialyllactose (Neu5Ac-α2,3-Gal-β1,4-Glc, from here on referred to as 3’SL) and α2,6-sialyllactose (Neu5Ac-α2,6-Gal-β1,4-Glc, from here on referred to as 6’SL) are also described. The structure of *Sp*CBM is compared to other known CBM40 structures showing that it shares a similar fold. In contrast to the other CBM40 domains, *Sp*CBM has a deep water-filled pocket adjacent to the N-acetyl moiety of sialic acid and surrounded by a positively charged surface. In the crystal structures, the acidic pocket is partially occupied by a lysine residue from a symmetry-related molecule. The structure of *Sp*CBM suggests that it may recognise a second receptor that may be responsible for the induction of chemokines, and the BBB invasion event.

## Results

### Structure overview

**Fig. 2:**
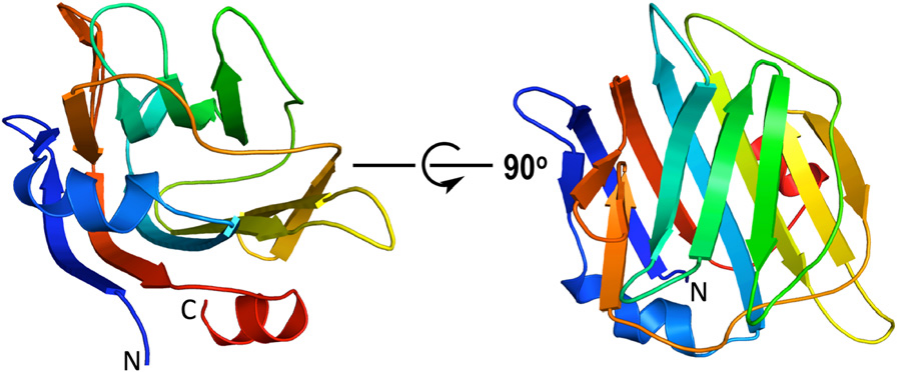
Overall structure of the *Sp*CBM. Cartoon representation of the *Sp*CBM in two orientations, coloured in rainbow colours from blue at the N-terminus to red at the C-terminus

**Fig. 3:**
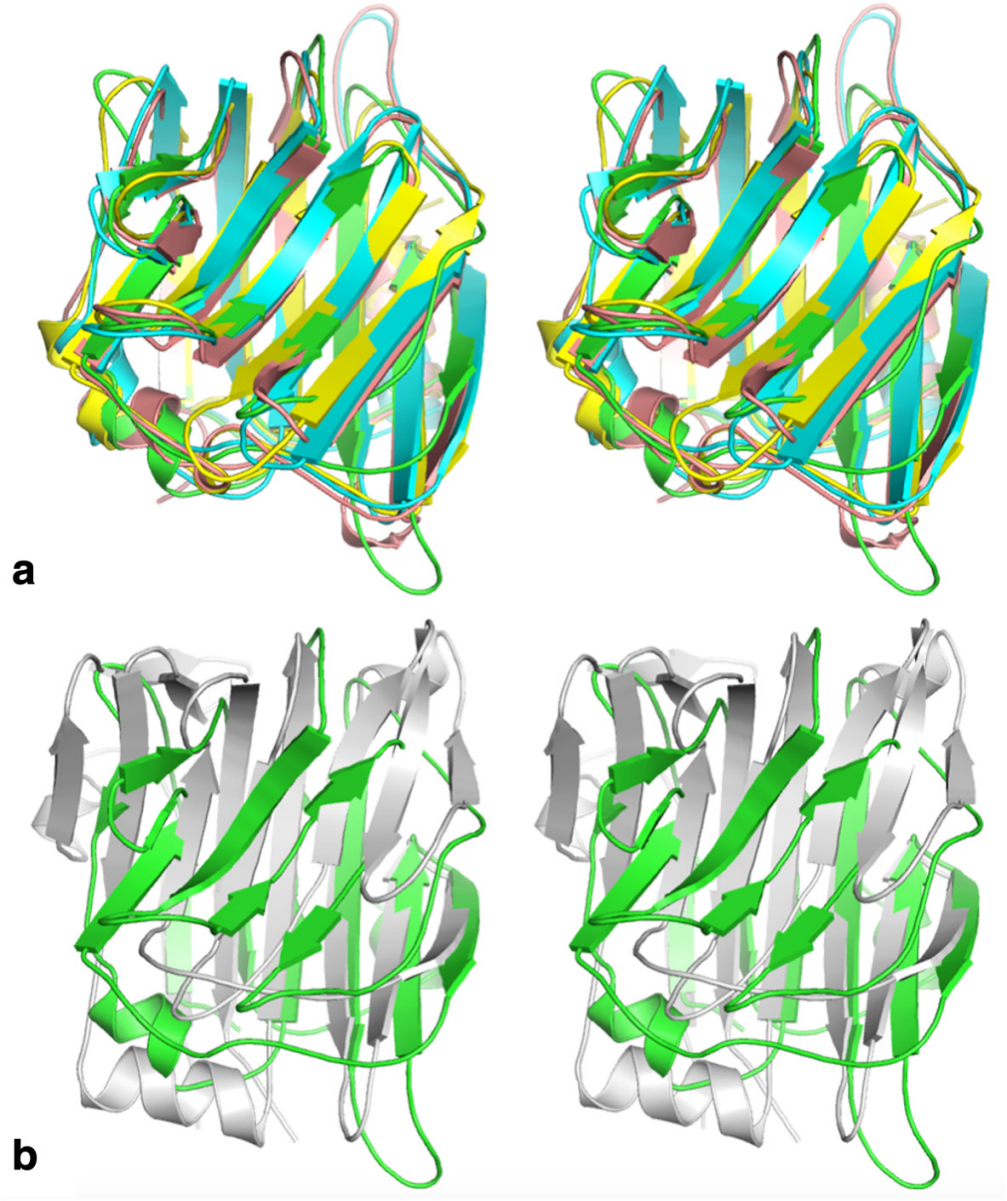
Stereo view of the cartoon representation of the superimposition of *Sp*CBM with other CBM40 family members. **a** *Sp*CBM is shown in green, NanB-CBM is shown in blue, NanJ-CBM is shown in yellow and NanL-CBM is shown in pink. **b** Superimposition of *Sp*CBM with NanH-CBM. NanH-CBM is shown in grey

All residues (from Val121 to Ser305) of the expressed CBM were clearly identifiable in the *Sp*CBM crystal structure. As shown in Fig. 2, the native structure constitutes a central β-sandwich (comprising one antiparallel β-sheet containing five-strands and a second antiparallel β-sheet containing six-strands), a β-hairpin on one side of the sandwich, an α-helix present between the β-sheets and a C-terminal α-helix packing against the β-sandwich. When compared to other CBM40 structures, *Sp*CBM is structurally homologous to CBM40 modules from *C. perfringens* NanJ [PDB ID: 2 V73]; RMSD 1.7 Å for 169 Cα’s [17], *S. pneumoniae* NanB [PDB ID: 2VW0]; RMSD 2.0 Å for 170 Cα’s [18], *M. decora* NanL [PDB ID: 2SLI]; RMSD 2.0 Å for 176 Cα’s [19], and *V. cholerae* NanH [PDB ID: 1W0P]; RMSD 3.1 Å for 134 Cα’s [20]. The superposition of *Sp*CBM with these structurally homologous domains is shown in Fig. 3.

### Ligand binding site

**Fig. 4:**
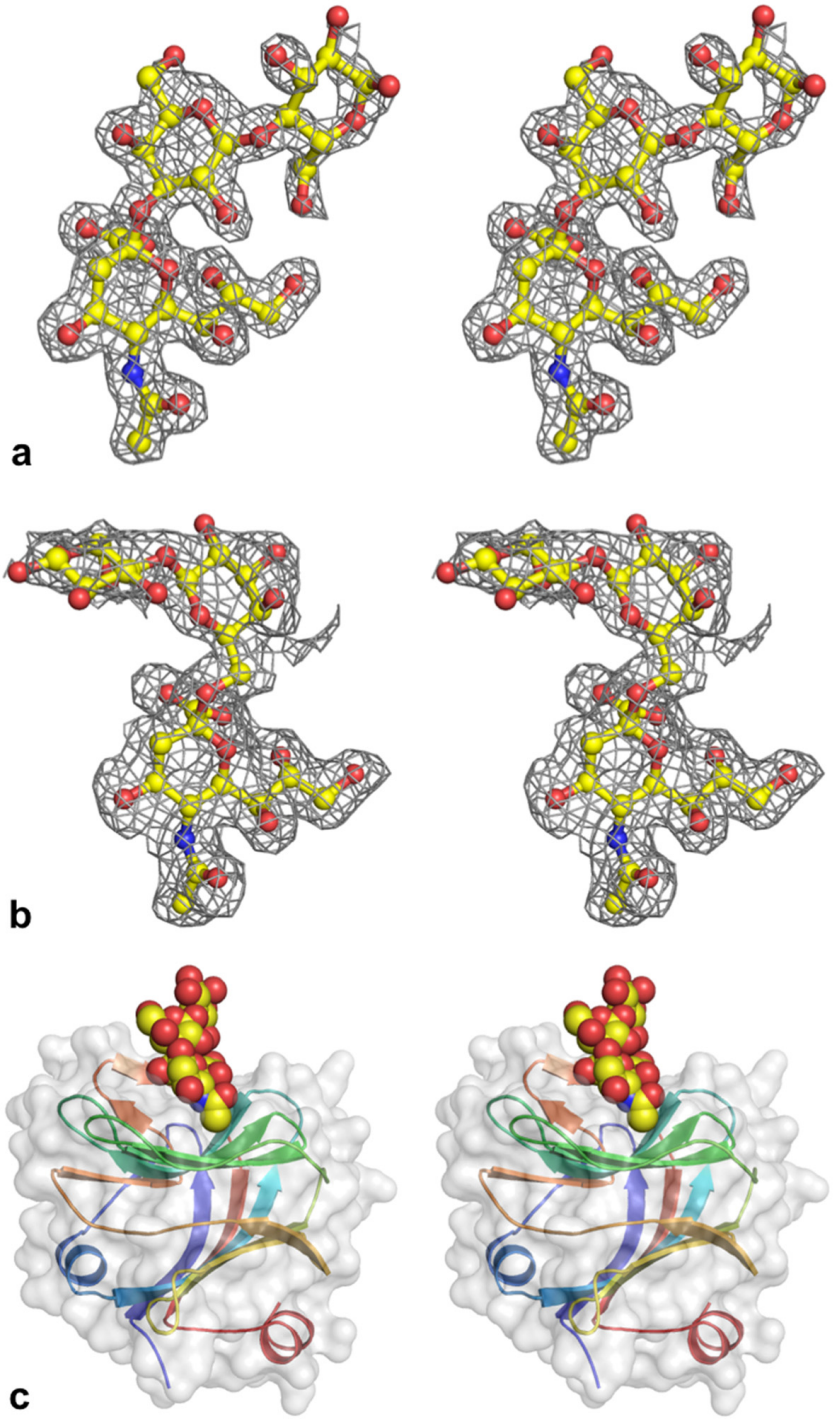
3’SL and 6’SL co-complexed with *Sp*CBM. **a** Stereo view of F_o_-F_c_ map of 3’SL co-complex contoured at 2.5σ level. **b** Stereo view of F_o_-F_c_ map of 6’SL co-complex contoured at 2.5σ level. **c** Stereo view of 3’SL (shown in spheres) bound to the *Sp*CBM substrate-binding site

For the complexed structures, both 3’SL and 6’SL were co-crystallized with *Sp*CBM. There are no significant conformational changes between the apo *Sp*CBM structure and the complexed structure, and superimposition of the apo and holo structures gives an RMSD of 0.48 Å over all Cα atoms for both 3’SL and 6’SL. Difference electron-density maps of both 3’SL and 6’SL molecules, in complex with *Sp*CBM, are clearly defined (Fig. 4), particularly the sialic acid moiety of each sialoside. In the 3’SL and 6’SL complexes, the protein structures are highly similar, giving an RMSD of 0.06 Å for all of the Cα atoms while the sialic acid moieties of the ligands are completely superimposable. The lactose moieties of the 3’SL and 6’SL, however, point in opposite directions (Fig. 4a & b). In 3’SL, lactose does not interact with the protein, although O6 of the glucose moiety interacts with O9 of sialic acid. In 6’SL, glucose O6 interacts with the side chain of Asp180. For both ligands, the lactose B-factors are significantly higher than the corresponding sialic acid moiety.

**Fig. 5:**
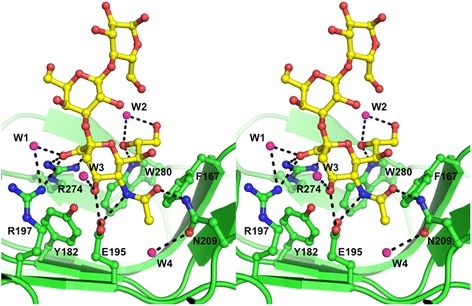
Stereo view of Neu5Ac binding sites of *Sp*CBM. 3’SL is shown in yellow carbon atoms and waters are shown in magenta. *Sp*CBM residues that are involved in the interaction are shown in stick representation with green carbon atoms. Hydrogen bonds are shown as black dotted lines

For *Sp*CBM, the residues that are involved in the interaction are mainly donated from the concave surface formed by the β-hairpin and three β-strands (Fig. 4c). As shown in Fig. 5, the carboxylate group of Neu5Ac forms a bidentate interaction with Arg274, a common feature in proteins that bind sialic acid. Arg197 also interacts with one of the carboxylate oxygens of Neu5Ac. Other Neu5Ac atoms make additional interactions with *Sp*CBM: O4 interacts with the side chain of Glu195 as does the N5 of the acetamido group; the O10 carbonyl oxygen of the acetamido group interacts with Asn209; the glycerol O8 hydroxyl oxygen interacts with the imidazole nitrogen of Trp280. Phe167 provides a hydrophobic platform supporting the glycerol carbons (C7 to C9) that are ~4 Å away, and also forms part of the hydrophobic pocket accommodating the acetamido methyl group. A number of water molecules are seen to bridge interactions between Neu5Ac and the protein. Details of the major interactions between *Sp*CBM and ligand are listed in Table 1.

**Table 1:** Interactions between *Sp*CBM and 3’SL. Intermolecular hydrogen bonds between *Sp*CBM and 3’SL are listed. Water molecules bound to 3’SL are also detailed

3’SL	Protein/Water atoms	Distance (Å)
O-1 A	Arg274-N-η2	2.88
O-1 A	Arg197-N-η1	3.15
O-1 A	H_2_O (W1)	2.74
O-1 B	Arg274-N-η1	2.90
O-4	Glu195-O-ε1	2.71
O-4	Glu195-O-ε2	3.39
O-4	H_2_O (W3)	2.66
O-8	Trp280-N-ε1	2.87
O-8	H_2_O (W2)	3.15
O-9	H_2_O (W2)	2.87
O-10	Asn209-N-δ2	2.79
N-5	Glu195-O-ε1	3.39
N-5	Glu195-O-ε2	2.77

### Acidic cavity adjacent to sialic acid binding site

**Fig. 6:**
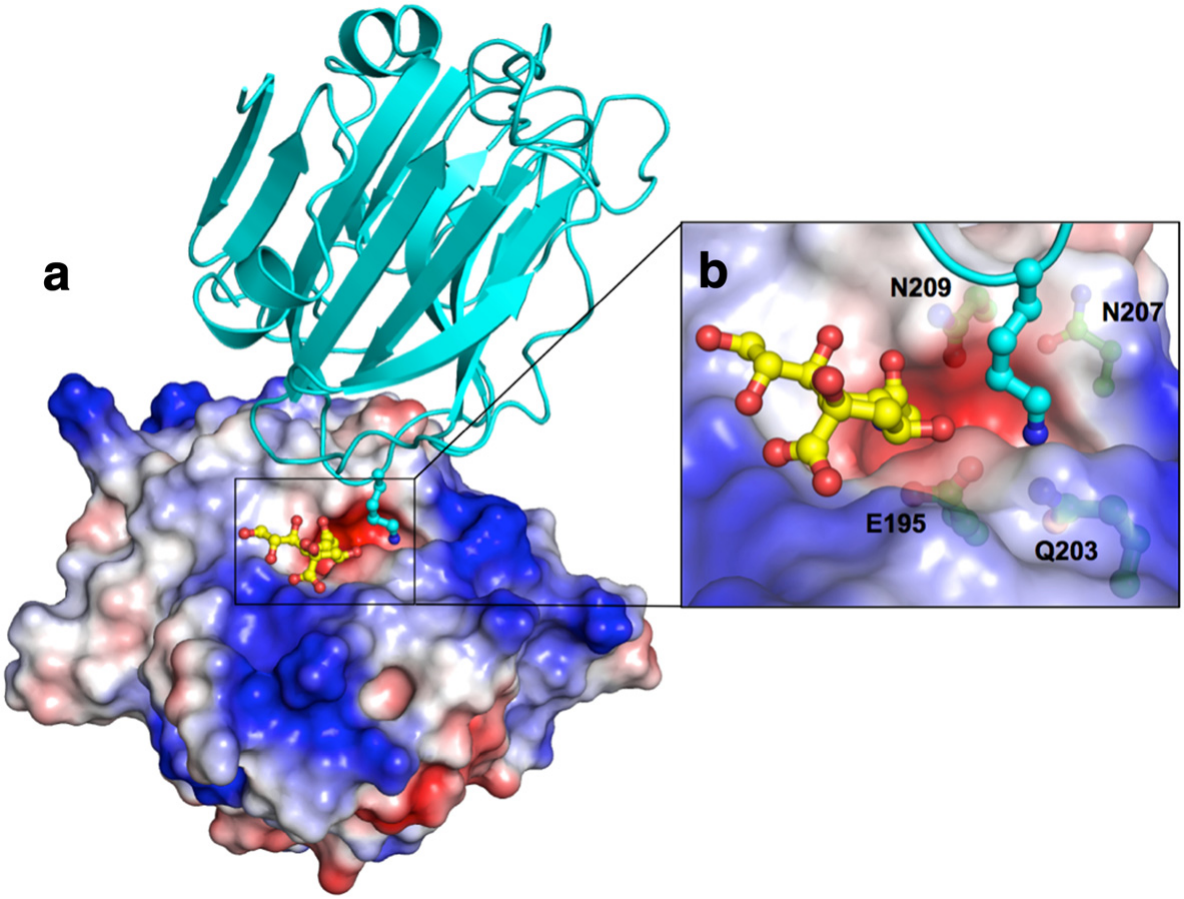
Potential functional cavity of *Sp*CBM. **a** The surface electrostatic view from the top of the binding site of *Sp*CBM and a cavity adjacent to the active site, which is occupied by a lysine from a symmetry related molecule (shown in cyan). **b** Close-up view of the interaction between the lysine and the protein. The solvent-accessible surface of *Sp*CBM is coloured based on the electrostatic potential from −7 (red) to +7 (blue*)* kT/e, calculated using the APBS tool in PyMOL [34, 35]

**Fig. 7:**
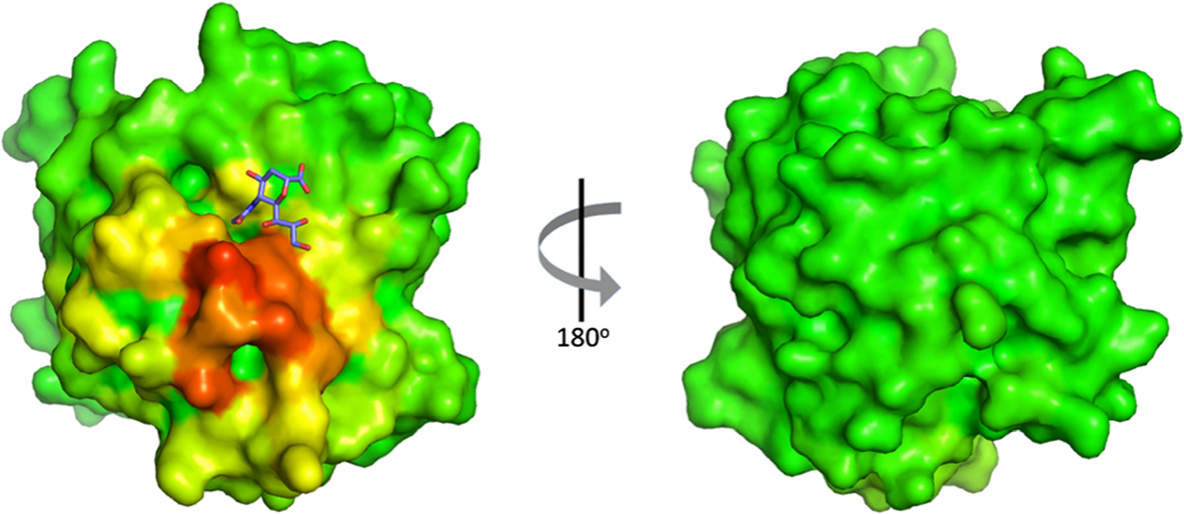
Prediction of protein-protein interaction site. The *Sp*CBM surface is coloured according to meta-PPISP score. Residues are coloured from green (low propensity for protein binding) to red (high propensity). The sialic acid moiety of 3’SL is shown in stick representation with purple carbon atoms

A striking feature of the protein is a deep, negatively charged cavity adjacent to the sialic acid binding site (Fig. 6a). This pocket is formed by four residues (Glu195, Gln203, Asn207 and Asn209), and is occupied by a lysine from a symmetry-related molecule (Fig. 6b). Interestingly, a region immediately adjacent to this pocket and to the sialic acid binding site is highlighted by the results of the meta-PPISP server as a likely protein interaction site (Fig. 7). No other protein binding sites on *Sp*CBM are predicted, and no such sites are predicted on the CBMs of NanB, NanL or NanH. NanJ CBM has a low-scoring patch in a region that overlaps with the corresponding patch on *Sp*CBM.

### Kinetics and binding affinity of *Sp*CBM interaction with 3’SL

**Fig. 8:**
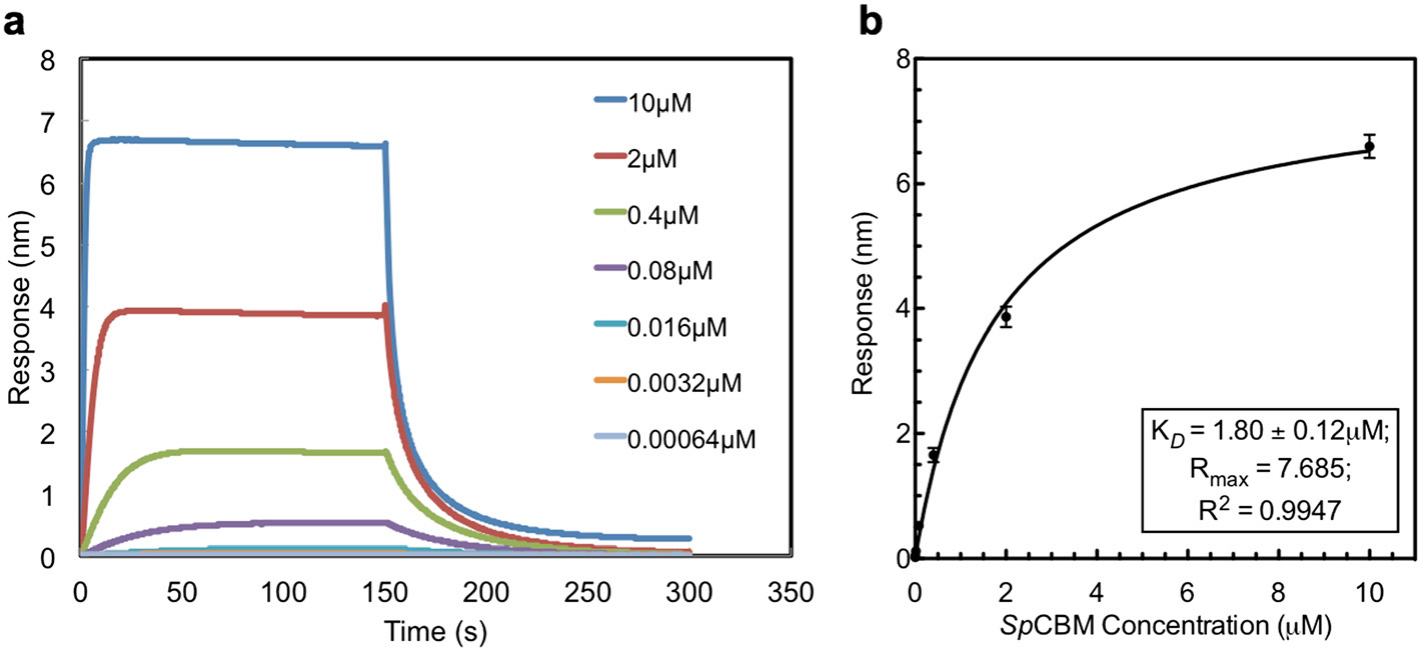
Bio-layer Interferometry. Association of *Sp*CBM with 3’SL immobilized to a probe via a biotin linkage as measured using bio-layer interferometry. **a** Sensogram showing the response units against increasing concentrations of *Sp*CBM. **b** Steady-state binding of *Sp*CBM-3’SL interaction determined by plotting the response at equilibrium against *Sp*CBM concentration and globally fitted using a 1:1 (Langmuir) binding model

A sensogram showing the association and dissociation of *Sp*CBM binding to 3’SL is shown in Fig. 8a. The kinetic parameters for the interaction based on global fitting of raw data using a 1:1 (Langmuir) binding model gave a K_*D*_ value of 1.8 ± 0.13 μM and R^2^ of 0.99 (data not shown). As equilibrium was also observed with the different *Sp*CBM concentrations, a K_*D*_ value of 1.8 ± 0.12 μM was determined from steady state binding by plotting the response at equilibrium against *Sp*CBM concentration (Fig. 8b).

## Discussion

CBM40 domains have been described structurally for other sialidases from *Vibrio cholerae* NanH [20], *Clostridium perfringens* NanJ [17], *S. pneumoniae* NanB [18] and *Macrobdella decora* NanL [19], with sialic acid binding only visualised in the first two. The CBM40 domains all share a common lectin β-sandwich fold with two antiparallel β-sheets containing five and six strands, in addition to other secondary structural elements. The sialic acid binding site is located in the concave surface of the five-stranded sheet, although the nature of the ligand interactions in the sialic acid complexes with *V. cholerae* NanH and *C. perfringens* NanJ are quite different and the binding sites are at different locations on the concave surface.

In their description of *C. perfringens* NanJ, Boraston *et al.* pointed out that there appear to be two subfamilies within the CBM40 family, one typified by *C. perfringens* NanJ and the other by *V. cholerae* NanH [17]. The structure of *Sp*CBM confirms that it belongs to the subfamily that includes *C. perfringens* NanJ and also *S. pneumoniae* NanB and *M. decora* NanL CBMs.

Previous reports mentioned that certain sialic acid binding residues from *C. perfringens* NanJ CBM (namely Glu79 and Arg81) were conserved in closely related CBMs from *S. pneumoniae* NanB and *M. decora* NanL but differed in NanA CBM. However, an alignment based on the *Sp*CBM structure reveals that these residues are, in fact, conserved and correspond to NanA residues Glu195 and Arg197. Other residues in the immediate area (Arg274 and Tyr182) are also conserved and form very similar interactions as Arg151 and Tyr66 in NanJ. In *S. pneumoniae* NanB, residues corresponding to NanA Glu195, Arg197, Arg274 and Tyr182 are conserved and adopt very similar positions to that of NanA, suggesting that NanB is likely to bind sialic acid in a similar manner.

The binding mode in the region of the glycerol moiety is somewhat different in NanJ CBM and *Sp*CBM. In *Sp*CBM, a hydrogen bond is formed between Trp280 and the C-7 hydroxyl, whereas the corresponding residue in NanJ (Tyr158) cannot make this interaction, instead forming a different H-bond to the glycerol group via Asn156. The space filled by this Asn side chain, along with the adjacent residue Tyr155, is not occupied by any residues in the corresponding area of *Sp*CBM or NanB, creating a more open binding pocket in the streptococcal proteins.

From binding affinity analysis, the dissociation constant, K_*D*_ for *Sp*CBM-3’SL interaction was found to be in the affinity range similar to that measured for the isolated CBM40 from *V. cholerae* NanH interacting with 3’SL (1.8 μM) as determined by Surface Plasmon Resonance (SPR) [21]. This suggests that the sialic acid binding pocket of both CBM40s may be similar. On examination of the residues involved in sialic acid binding by *Vc*CBM [20], both CBM40s involve a comparable number of direct and water-mediated interactions that target the sialic acid moiety alone, despite the overall topology of the binding sites being different between them.

Besides the classic function of binding to sialic acid, NanA was reported to enhance the *S. pneumoniae* interaction with human brain microvascular endothelial cells (hBMECs) via an adhesin function of NanA-CBM, which can potentially facilitate the entry of bacterial pathogens into the central nervous system (CNS), even with little contribution of the sialidase activity [10]. However, it is still not completely clear which receptor in the hBMECs is important for this process, and how it could be recognized by NanA-CBM. In the current study, the surface electrostatic view shows that there is a deep, negatively charged cavity with positively charged surface next to the sialic binding site (Fig. 6a), which is not present in the other family 40 CBMs. Of the four amino acids that form the cavity, only Glu195 is conserved, whereas Gln203, Asn207 and Asn209 are present in a region of sequence that exhibits low homology in family 40 CBMs, suggesting that this surface feature is exclusive to *S. pneumoniae* NanA. The four residues interact with a lysine from a symmetry related molecule (Fig. 6b). Therefore, it is possible that this region is important for *S. pneumoniae* NanA CBM interaction with a host cell receptor. This proposed interaction site for a binding partner for NanA CBM is supported by the output of the metaPPISP web server, which combines results from three different methods for predicting protein-protein interaction sites. The predicted region lies directly adjacent to the sialic acid binding site and the acidic pocket.

## Conclusions

In summary, we have determined the structure of the isolated form of CBM from *S. pneumoniae* NanA, which has been identified as a Family 40 CBM due to its ability to bind the terminal sialic acid of glycoconjugates. Our findings suggest that this domain may enhance the virulence of NanA by targeting and binding to a variety of linkage-independent sialic acid receptors that line the surface of respiratory epithelial cells. Further experiments to determine the NanA binding partner(s) are ongoing. The *Sp*CBM domain, in addition to showing promise as a bio-therapeutic against respiratory pathogens [12, 22], is a potential drug target and may be exploited as part of a combinatorial drug design approach to inhibit NanA attachment and catalysis.

## Methods

### Cloning, expression, and purification

The gene encoding the *Sp*CBM domain from *S. pneumoniae* NanA (UniProt: P62575) was generated by the polymerase chain reaction (PCR) using the following primers 5’-GGCT**CCATGG**TGATAGAAAAAGAAGATG-3’ and 5’-GCA**CTCGAG**TCATTTAAAAAGTTGACTACG-3’ (*Nco*I and *Xho*I restriction sites in bold) and pQE30 vector containing the *nanA* sialidase gene as template. The PCR fragment was purified using a Gel Extraction Kit (QIAGEN) prior to ligation into an appropriately digested pEHISGFPTEV vector [23]. The construct was propagated in *Escherichia coli* DH5α cells with positive colonies identified by colony PCR. The DNA sequence was confirmed by sequencing (The Sequencing Service, University of Dundee, UK), prior to transforming *E. coli* BL21 (DE3) expression strain (Novagen) for protein production.

Expression of *Sp*CBM was achieved by inoculating Luria Broth (LB) medium containing 50 μg/ml kanamycin with a single colony and incubating at 37 °C until cultures reached an absorbance at 600 nm (*A*_600_) of 0.6. Cultures were subjected to heat shock at 42 °C for 20 minutes prior to cooling to 25 °C and induced with isopropyl thio-β-D-galactopyranoside (IPTG, 0.5 mM final concentration) to induce expression of *Sp*CBM. Cultures were left to incubate further overnight at 18 °C before harvesting by centrifugation at 10,000 *g* for 20 min. Cell pellets were resuspended in phosphate-buffered saline (PBS; 20 mM sodium phosphate, 150 mM sodium chloride, pH7.4) containing 10 mM imidazole and 300 mM sodium chloride with DNase I (Sigma, final concentration 20 μg/ml) and EDTA-free protease inhibitor tablets (one tablet per 50 ml extract, Roche Diagnostics). The cell suspension was lysed by sonication to disrupt cells then subjected to centrifugation at 40,000 *g* for 30 min at 4 °C to remove cell debris. Clarified supernatants were collected and filtered with a 0.2 μm pore size syringe-driven filter before further protein purification.

The soluble cell extract was initially loaded onto a 20 ml HisPrep FF 16/10 column (GE Healthcare) equilibrated in PBS containing 10 mM imidazole and 300 mM sodium chloride. The column was then washed with PBS buffer containing 20 mM imidazole and 300 mM NaCl before eluting bound protein using the PBS/NaCl buffer supplemented with 250 mM imidazole. Eluted fractions were then treated with TEV protease overnight to remove the His-GFP tag. Cleaved proteins were further purified by re-applying to the 20 ml HisPrep FF 16/10 column. The collected flow through was concentrated before performing size exclusion chromatography using a HiPrep 26/60 Sephacryl S-200 HR column (GE Healthcare), which was pre-equilibrated in 20 mM sodium citrate, pH6.0 containing 50 mM sodium chloride. Fractions from the observed peaks were analyzed separately by SDS-PAGE gel. Protein identity and integrity were confirmed by mass spectrometry (BSRC Mass Spectrometry and Proteomics Facility, University of St Andrews). Purified *Sp*CBM was collected, concentrated and stored at −80 °C for future use.

### Bio-Layer Interferometry (BLI)

The binding affinity assay of *Sp*CBM to 3’SL was performed using the ForteBio Octet RED384 system (ForteBio). Assays were performed in black 96 wells plates (Nunc™ F96 MicroWell™ plate, Thermo Scientific) using PBS containing 0.002 % Tween-20 as running buffer at 25 °C. Super streptavidin-coated (SSA) biosensor tips (ForteBio) were pre-hydrated in 200 μl running buffer for 10 min followed by equilibration in PBS for 60 s. Tips were non-covalently loaded with a 25 μg/ml solution of a multivalent biotinylated 3’SL-polyacrylamide (Glycotech) in running buffer for 300 s followed by a wash of 60 s in the same buffer. All sensors, including reference sensors (no ligand), were blocked with biocytin (Life Technologies) for 60 s, to prevent non-specific interactions of protein to the sensor surface, followed by a further wash for 60 s. Association of biotinylated ligand with *Sp*CBM (5-fold dilution series using a 10 μM stock in running buffer) was performed for 150 s before dissociation of binding was performed using running buffer for 150 s. All experiments were performed in triplicate. Data were processed to calculate kinetic and affinity parameters using the ForteBio software.

### Protein crystallization

Purified *Sp*CBM was concentrated to 33 mg/ml based on the results of precrystallization assay kit screening (Hampton Research). All the subsequent crystallization experiments were done at 20 °C by the sitting-drop, vapour-diffusion method. Initially, commercial kits Crystal Screen, SaltRx, Index (Hampton Research), Wizard, Cryo I&II (Emerald BioSystems), JCSG Suite, PACT Suite and PEGs Suite (Qiagen) were screened by a Honeybee 963 robot system (Genomic Solutions) for protein crystallization. Conditions with crystalline materials were selected for crystallization optimization. After several rounds of optimizations, the best crystals were obtained in 120 mM MMT (molar ratios 1:2:2 of DL-malic acid: MES: Tris Base) buffer pH9.0, 25 % (w/v) PEG1500. Crystals appeared the next day and reached their maximum size within two weeks. Structures of complexes with Neu5Ac derivatives were obtained by co-crystallization with *Sp*CBM. Protein solution (33 mg/ml) containing 5 mM ligand was incubated at 4 °C for 30 min followed by mixing with an equal volume of reservoir solution (100 mM MMT pH9.0 and 28 % (w/v) PEG1500). The crystals appeared the next day and reached maximum size in 3–4 days.

### X-ray diffraction data collection and processing

Crystals were cryoprotected by transfer for a few seconds to a solution of the crystallization buffer with 5 % (w/v) ethylene glycol added before data collection at 100 K. All X-ray diffraction data were collected in-house on a Rigaku 007HFM (Cu anode, λ = 1.54178) X-ray generator, with a Saturn 944CCD detector. HKL2000 was used for data processing and integration [24]. Apo *Sp*CBM crystals belong to the monoclinic space group *P*2_1_, with two monomers in an asymmetric unit. Crystals of complexes were also *P*2_1_ but contained one monomer per asymmetric unit. Data collection statistics are given in Table 2.

**Table 2:** Data collection and refinement statistics

Crystal	Apo	3’SL complex	6’SL complex
Data collection^a^			
Space group and cell dimensions (Å,°)	P2_1_	P2_1_	P2_1_
	a = 39.2, b = 67.0, c = 66.8, β = 92.3	a = 42.6, b = 44.5, c = 43.0, β = 97.0	a = 42.5, b = 44.6, c = 43.0, β = 97.0
Resolution (Å)	67.0-1.8	44.5-1.8	44.6-1.8
Unique reflections	29,236	12,926	13,634
Completeness (%)	97 (74)	92 (76)	97 (83)
Redundancy	2.5 (2.0)	2.4 (1.7)	3.0 (2.2)
R-merge^b^	0.031 (0.069)	0.069 (0.108)	0.049 (0.093)
I/σI	43.9 (22.7)	38.7 (16.8)	44.1 (16.8)
Refinement			
Reflections used	27,743	12,277	12,950
Number of protein atoms	2,986	1,532	1,528
Number of ligand atoms	-	43	43
Number of waters	403	166	148
R-factor^c^	0.163	0.143	0.150
R-free^d^	0.218	0.198	0.195
rmsd bond lengths (Å)	0.02	0.02	0.02
rmsd bond angles (°)	2.10	2.04	2.08
Average B-factors (Å^2^)			
all atoms	16.3	15.2	15.9
ligand atoms	-	27.8	32.4
waters	28.5	27.8	26.4
Molprobity score	1.80	1.20	1.40
Ramachandran favoured/outliers (%)	97/0	98/0	97/0

^a^Numbers in parentheses refer to the highest resolution shell

^b^R-merge = Σhkl Σi | Ihkl, i - < Ihkl > | / Σhkl < Ihkl>

^c^R-factor = (Σ | |Fo| - |Fc| |) / (Σ |Fo|)

^d^Test set comprised 5 % of reflections

### Structure determination and refinement

The leech *trans*-sialidase structure [PDB ID: 2SLI] was used to solve the *Sp*CBM structure by molecular replacement with the PHASER program from the CCP4 suite [25, 26]. Refinement was carried out with the program REFMAC5 [27] from the CCP4 suite and the refined model was manually adjusted in Coot [28]. After further refinement with REFMAC5, the structures were inspected and validated with Coot and MolProbity [29]. Refinement statistics are summarized in Table 2.

### Prediction of protein-protein interaction sites

The meta-PPISP server was used to predict potential protein-protein interaction sites on *Sp*CBM [30]. The server combines three different methods in a linear regression analysis, a strategy which improves accuracy compared to the individual methods [31–33]. For comparison, the same analysis was carried out on the other family 40 CBMs of known structure.

## Availability of supporting data

Coordinates and structure factors have been deposited in the Protein Data Bank with accession numbers 4ZXK, 4C1W and 4CIX for the apo structure, the 3’SL complex and the 6’SL complex, respectively.

## Abbreviations

NanA: Neuraminidase A
CBM: Carbohydrate-Binding Module
*Sp*CBM: *Streptococcus pneumoniae* NanA CBM
3’SL: α2,3-sialyllactose
6’SL: α2,6-sialyllactose
hBMECs: human brain microvascular endothelial cells
BBB: Blood–brain-barrier
RMSD: Root Mean Square Deviation
SPR: Surface Plasmon Resonance
BLI: Bio-Layer Interferometry
