# Erratum to: Structural characterization of the carbohydrate-binding module of NanA sialidase, a pneumococcal virulence factor

**DOI:** 10.1186/s12900-015-0047-z

**Published:** 2015-10-06

**Authors:** Lei Yang, Helen Connaris, Jane A. Potter, Garry L. Taylor

**Affiliations:** Biomedical Sciences Research Complex, University of St Andrews, St Andrews, KY16 9ST Fife, UK

After publication of this article [1], it was noticed there was an error in the Methods section under the subsection: Availability of supporting data.

The text including the error is as follows: “Coordinates and structure factors have been deposited in the Protein Data Bank with accession numbers 4ZXK, 4C1W and 4CIX for the apo structure, the 3^1^SL complex and the 6^1^SL complex, respectively.” Instead of 4CIX, it should read 4C1X.

This error has since been updated in the article [1].
